# Design and Implementation of an Omni-Directional Underwater Acoustic Micro-Modem Based on a Low-Power Micro-Controller Unit

**DOI:** 10.3390/s120202309

**Published:** 2012-02-20

**Authors:** Tae-Hee Won, Sung-Joon Park

**Affiliations:** Digital Communications Laboratory, Electronic Engineering, Gangneung-Wonju National University, Gangneung, Gangwon 201-702, Korea; E-Mail: tieits@nate.com

**Keywords:** underwater communication, underwater wireless sensor network, underwater acoustic modem, micro-modem, acoustic wave, Cortex-M3, omni-directional transducer

## Abstract

For decades, underwater acoustic communication has been restricted to the point-to-point long distance applications such as deep sea probes and offshore oil fields. For this reason, previous acoustic modems were typically characterized by high data rates and long working ranges at the expense of large size and high power consumption. Recently, as the need for underwater wireless sensor networks (UWSNs) has increased, the research and development of compact and low-power consuming communication devices has become the focus. From the consideration that the requisites of acoustic modems for UWSNs are low power consumption, omni-directional beam pattern, low cost and so on, in this paper, we design and implement an omni-directional underwater acoustic micro-modem satisfying these requirements. In order to execute fast digital domain signal processing and support flexible interfaces with other peripherals, an ARM Cortex-M3 is embedded in the micro-modem. Also, for the realization of small and omni-directional properties, a spherical transducer having a resonant frequency of 70 kHz and a diameter of 34 mm is utilized for the implementation. Physical layer frame format and symbol structure for efficient packet-based underwater communication systems are also investigated. The developed acoustic micro-modem is verified analytically and experimentally in indoor and outdoor environments in terms of functionality and performance. Since the modem satisfies the requirements for use in UWSNs, it could be deployed in a wide range of applications requiring underwater acoustic communication.

## 1. Introduction

Underwater acoustic communication is a technique which enables devices to exchange data wirelessly in the water. Since the radio frequency (RF) commonly used for terrestrial communication networks is not transmitted very far in a water medium due to severe absorption and scattering, sound and ultrasonic waves having frequencies under 100 kHz are typically used as a carrier [1,2]. For several decades, the main applications of underwater acoustic communication have been limited to deep sea probes and exploitation of offshore oil fields, requiring acoustic modems which consume high power but support long working ranges [3,4].

Quite recently, underwater wireless sensor networks (UWSN) have arisen as a promising field in research and development [5–7], driven by the expansion of applications including data collection and pollution monitoring for the conservation of ecological systems in fresh water and sea water, tactical surveillance, data transfer for unmanned underwater vehicles, and other scientific purposes. Some initial studies on small-scale underwater modems have been described in [8–11]. The first low-power modem, named CORAL [8], comprised a micro-controller unit (MCU), analog circuitry and piezo-transducer having a resonant frequency of 1.7 kHz. The modem was tested in a small water tank and verified one-way communication at the distance of 20 cm. Another low-power modem developed by Will *et al*. [9] was equipped with speaker and microphone and only tested in air environment. In both acoustic modems, transmission data rates and bit error probability were not reported. In [10,11], we implemented bidirectional acoustic modems equipped with piezo-transducers and verified a working distance of 30 m and a data rate of 5 kbps in a pond. The abovementioned early stage modems are meaningful from the viewpoint that they verified the feasibility of compact and low-power acoustic modems, however they could not be utilized in real underwater applications since they were equipped with piezo-transducers whose use is generally restricted to onshore communication systems.

One full-scale modem for UWSNs was developed by the Woods Hole Oceanographic Institution (WHOI), and was called a micro-modem in [12]. The dimensions of base board are 114 mm and 44 mm in length and width, and the whole modem consists of a stack of boards. The modem supports a variety of data rates, ranging from 80 to 5,400 bps depending on the power consumption that ranges from 50 W to 100 W. Tritech Corporation has also produced a commercial acoustic micro-modem whose dimensions are 54 by 74 mm in diameter and height, respectively. The modem supports a working range of 500 m with a power consumption of 7.2 W, but its data rate is merely 40 bps [13]. Since these two micro-modems described above are equipped with underwater transducers having cylindrical shapes and low resonant frequencies between 10 and 30 kHz, they have toroidal beam pattern in the vertical axis and the size of their transducers is quite large.

Meanwhile, in order to apply an acoustic modem to UWSNs, it has to possess several properties, including low-power consumption, omni-directional beam pattern, small size, low cost, moderate data rate and good working range. From these observations, in this paper we design and implement a micro-modem satisfying all these requirements based on our previous work [14,15]. As a transducer to be interlocked with our micro-modem, a spherical omni-directional transducer having a resonant frequency of 70 kHz and a diameter of 34 mm is considered. Physical layer frame format and symbol structure are also studied to construct an efficient packet transmission system. The implemented modem was tested in a water tank, a pond and a river to verify its functional operations and performance.

The remainder of this paper is organized as follows: in Section 2, we describe the phenomenological issues occurring during data transfer in underwater channels, especially in terms of transmission loss and transducer loss. The design issues of our acoustic micro-modem are explained in Section 3, and digital domain processing and analog domain processing are described in detail in Section 4 and Section 5, respectively. The implemented micro-modem is shown with actual images in Section 6. Experimental environments and results in a water tank, a pond and a river are provided and analyzed in Section 7. Finally, in Section 8, we provide a brief summary of this paper.

## 2. Data Transfer in Underwater Channels

### 2.1. Transmission Loss

Acoustic waves emitted from a transducer undergo an attenuation in signal strength as they propagate. According to [2], if spherical spreading is assumed, the transmission loss (*TL*) of acoustic waves becomes:

(1) TL=20 log d+α⋅d

where *d* represents the distance between wave source and destination and *α* means the absorption coefficient depending mainly on frequency. Also, if cylindrical spreading is assumed, *TL* becomes:

(2) TL=10 log d+α⋅d

where transmitted acoustic waves are trapped between two parallel and perfectly reflecting surfaces.

**Figure 1.**
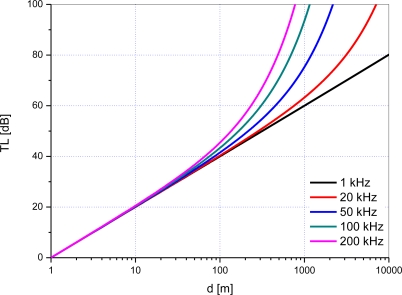
Transmission loss with respect to communication distance and frequency.

Figure 1 shows *TL* with respect to *d* and frequency in spherical spreading and sea water with the assumptions that the temperature is 10 degrees Celsius, salinity is 35 per mil, and pH is 7.

From the results, it is observed that the transmission loss on a dB scale is nearly proportional to the logarithm of distance up to 100 m. In other words, the second term of the right side in Equation (1) is negligible due to the relatively small value of the absorption coefficient. Meanwhile, when the distance exceeds around 100 m, the transmission loss increases exponentially due to the second term. Acoustic waves having high frequency are especially susceptible to absorption with the increase of distance and thus they experience large transmission loss gradually.

### 2.2. Transducer Loss

The sonar equations describe the relationship between sound pressures and voltages at a transducer for underwater acoustic communication [16]. When an input voltage *V_in_* is applied to a transmitting transducer, the sound pressure level *SPL_T_* is generated at the transducer, which is given by:

(3) $${\mathrm{SPL}}_{T}=\mathrm{TRV}+20\;\text{log}\;V_{\mathrm{in}}$$

where *TRV* is the transmitting response to the input voltage which is a characteristic value for each transducer. Due to the transmission loss through an underwater channel, the sound pressure level at a receiving transducer *SPL_R_* becomes:

(4) $${\mathrm{SPL}}_{R}={\mathrm{SPL}}_{T}-\mathrm{TL}$$

At the receiving transducer, *SPL_R_* is converted into the output voltage *V_out_* which is given by:

(5) $$20\;\text{log}\;V_{\mathrm{out}}={\mathrm{SPL}}_{R}+\mathrm{RR}$$

where *RR* is the receiving response of the transducer. Equations (3), (4) and (5) can be combined and rewritten as follows:

(6) $$20\;\text{log}\;\frac{V_{\mathrm{out}}}{V_{\mathrm{in}}}=\mathrm{TRV}+\mathrm{RR}-\mathrm{TL}$$

In Equation (6), −*TRV* + *RR* can be interpreted as the transducer loss specific to each transducer.

## 3. Overview of Underwater Acoustic Micro-Modem

**Figure 2.**
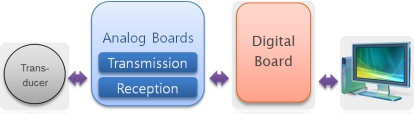
Block diagram of our micro-modem.

Figure 2 illustrates the block diagram of the overall structure of our underwater acoustic micro-modem. The modem consists of a digital board, two analog boards and a transducer and it can be interconnected with external devices such as personal computer (PC) by the universal asynchronous receiver and transmitter (UART) protocol.

For data transmission, the digital board gets information data from the PC, makes a physical layer frame and delivers it to the analog transmission board. At the analog transmission board, a modulated signal is generated and then amplified. The electric signal is transformed to acoustic waves at the transducer and sent through an underwater channel. On the contrary, when acoustic waves arrive at the transducer, they are converted to electric signals. After signal amplification and detection at the analog reception board, the estimates of information data are reproduced at the digital board and are transferred to a monitoring terminal.

## 4. Digital Domain Processing

### 4.1. MCU

The ARM Cortex-M3 processor is the industry-leading 32-bit processor for highly deterministic real-time application embedding microcontrollers such as home electronic appliances, industrial control systems and wireless sensor networking systems. The processor is built on a high performance 3-stage pipeline Harvard architecture core, making it possible to simultaneously occupy a significantly reduced physical area and possess exceptional power efficiency.

For the implementation of the digital domain algorithms of our micro-modem, the MCU STM32F103ZE containing Cortex-M3 processor was chosen. The main features of the MCU are as follows: When compared to the ATmega128 mounted on our previous modem [14], the STM32F103ZE supports faster processing speed, larger memory and peripherals and smaller chip size which enable the modem to handle signal generation and detection algorithms in the digital domain.

- Core: 32-bit ARM Cortex-M3 CPU
- Processing speed: 1.23 Dhrystone MIPS
- SRAM: 64 Kbytes
- Flash memory: 512 Kbytes
- Three 12-bit ADC, two DAC
- Four general-purpose 16-bit timers, two PWM timers
- Three SPI, five USART, two I^2^S

### 4.2. Digital Signal Processing

**Figure 3.**
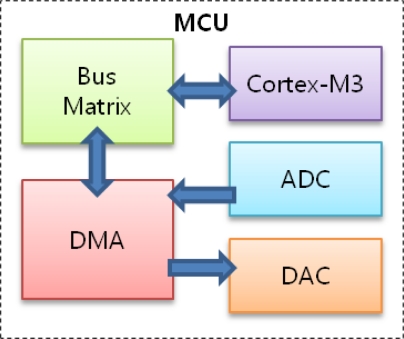
Block diagram of MCU.

When the modem is powered on, the MCU initializes peripheral registers. After initialization, the system remains idle, waiting for interrupts from the PC via UART or an analog reception board via an analog-to-digital converter (ADC). If the MCU receives data from the PC, it carries out predetermined physical layer functions on the data and transfers the outputs to a digital-to-analog convertor (DAC) to generate sinusoidal waves for transmission. After completion, it returns to idle mode. On the other hand, if the MCU receives an interrupt from the ADC which indicates that an acoustic signal hass arrived at the receiving transducer, it starts to sample the signal. The sampling frequency is set to 1 MHz and the number of bits for quantization in the ADC is fixed at 12. After the MCU performs appropriate functions on the sampled data, it delivers the outputs to the PC. Figure 3 depicts the block diagram explaining the procedures in the MCU. Here the DMA is used to fetch data from the ADC and hand over data to the DAC without the help of the Cortex-M3.

### 4.3. Physical Layer Frame Format

**Figure 4.**
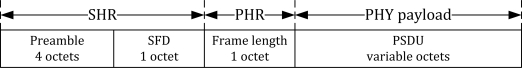
Physical layer frame format.

For the hierarchical data transmission in underwater packet-based communication systems, the frame format of the physical layer is designed as depicted in Figure 4. At the beginning of each frame, a preamble having a length of 4 octets and the value 0xAAAAAAAA is inserted to recognize the existence of a frame and obtain symbol synchronization at the receiver. The following field is SFD, indicating the end of the preamble. In this application, one octet is allocated for SFD and its value is 0xFF. The preamble and SFD are often called a synchronization header (SHR) because they are used for frame and symbol synchronization. After SHR, a frame length field is inserted to inform a receiver of the length of physical layer payload. Frame length field itself constitutes the physical layer header (PHR). The last field is the physical layer service data unit (PSDU). Since the length of the frame length field is one octet, the maximum length of PHY payload amounts to 255 octets. All of the abovementioned field types and lengths could be varied according to the underwater channel environment, maximum data rate of the acoustic modem, and desired underwater applications.

## 5. Analog Domain Processing

### 5.1. Analog Signal Processing at Transceiver

**Figure 5.**
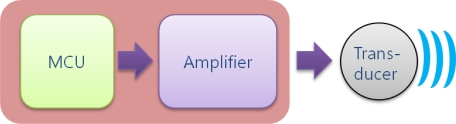
Functional diagram of the transmitter.

The signal flow for data transmission is depicted in Figure 5. As shown, the main objective of analog signal processing is amplification with high power. The output voltage of the MCU after DAC is limited to 3 volts, however, by passing through the amplifier, an analog signal having a maximum 200 volts peak-to-peak is generated and applied to the transducer. The amplifier module is made up of power amplifier ICs and a transformer.

**Figure 6.**
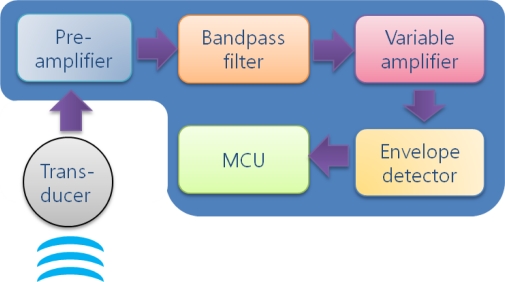
Functional diagram of receiver.

The functional diagram of a receiving modem is shown in Figure 6. Once acoustic waves detected at the transducer are transformed into electric signals, analog signal processing is conducted by passing in order through a pre-amplifier, bandpass filter, variable amplifier and envelope detector. Since the electric signal output from the transducer has a very low signal level on the order of a few millivolts, it is primarily amplified at the pre-amplifier with tens of amplification gain. Then, as the realization of non-coherent detection, bandpass filtering and envelope detection are conducted about the resonant frequency. In between them, a variable amplifier which is implemented by an analog IC is inserted in order to maintain the signal level steady for the envelope detection to be properly performed. It is possible to change the amplifier gain into one of the four gain values (1, 2, 4 and 8) with the help of a controlling signal from the MCU. Finally, the analog signal is supplied to the MCU for ADC and the reconstruction of data.

### 5.2. Modulated Symbol Structure

Data sent through underwater channels undergo severe attenuation and distortion by multipath propagation and time-domain channel variation. Thus, it is very difficult to acquire the exact phase of each symbol at a receiving modem in such a harsh environment. Thus, since acoustic micro-modems should possess the properties of small size, low power consumption and low cost, amplitude shift keying with non-coherent detection which does not require the phase of signal for detection is chosen for the modulation and demodulation of this research.

**Figure 7.**
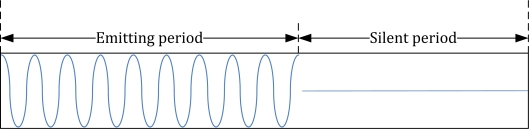
Symbol structure for the transmission of 1.

Meanwhile, in order to mitigate the intersymbol interference caused by multipath propagation, each modulation symbol is comprised of an emitting period and a silent period. The silent period is a kind of symbol-level guard time where no signals are transmitted. That is, for the transmission of ‘1’, sinusoidal electric signals are applied to a transducer during the emitting period whereas no signals are generated for the silent period as illustrated in Figure 7. For the transmission of ‘0’, no signals are generated for the entire symbol duration since amplitude shift keying is utilized.

## 6. Implemented Micro-Modem

**Figure 8.**
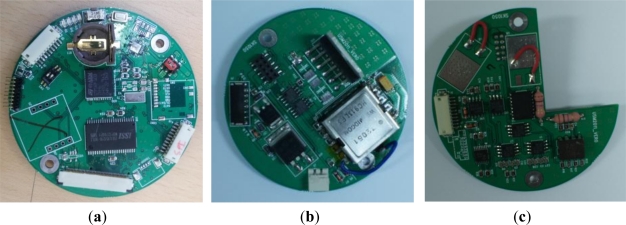
(**a**) Digital board; (**b**) Analog transmission board; (**c**) Analog reception board.

The building blocks of our micro-modem developed by the abovementioned design methodology are shown in Figure 8. The subfigures represent the digital board, analog transmission board and analog reception board, respectively.

**Figure 9.**
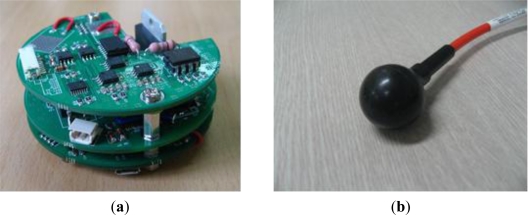
(**a**) Modem hardware; (**b**) Omni-directional transducer.

The three boards are assembled and stacked to constitute the modem hardware as shown in Figure 9(a). The bottom layer is the digital board which is responsible for digital signal processing. It is equipped with SPI and UART ports to interwork with other devices and a regulator module to generate 3.3 volts.

The middle layer is the analog transmission board. Besides the circuitry of analog signal processing for transmission, it is also equipped with a regulator producing 12 volts in order to supply power to several analog elements. The top layer is the analog reception board in charge of analog signal processing for reception.

Figure 9(b) shows the spherical transducer to be connected to the modem hardware shown in Figure 9(a). The transducer has a resonant frequency of 70 kHz and a diameter of 34 mm. Owing to its spherical structure, it provides an omni-directional beam pattern tridimensionally. Its *TRV* is 147 dB re 1 uPa/V at 1 m and *RR* is −200 dB re 1 V/uPa. The maximum input power is restricted to 190 W for normal operation.

The implemented micro-modem is operated by an external general-purpose lithium-ion battery of 14.8 volts, consuming 4.5 watts. As mentioned before, the supply voltage is converted to 12 volts and 3.3 volts through regulators for the operation of two analog boards and the digital board, respectively.

## 7. Experimental Results

### 7.1. Indoor Test

**Figure 10.**
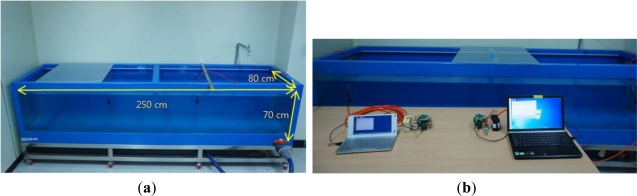
Experimental environment for the tests in a water tank. (**a**) Water tank; (**b**) System setup.

In order to verify the functionality and performance of the developed micro-modem, indoor experiments were conducted in a water tank whose dimensions are 250 cm, 80 cm and 70 cm in length, width and height, respectively, as shown in Figure 10(a). For the sake of convenience, transducers are only submerged in the water medium and the rest of micro-modem is placed on the desk in front of the water tank as shown in Figure 10(b). A transducer and a modem are connected by a direct cable. Also, each modem is connected to a corresponding computer via RS-232 cable for control and monitoring.

**Figure 11.**
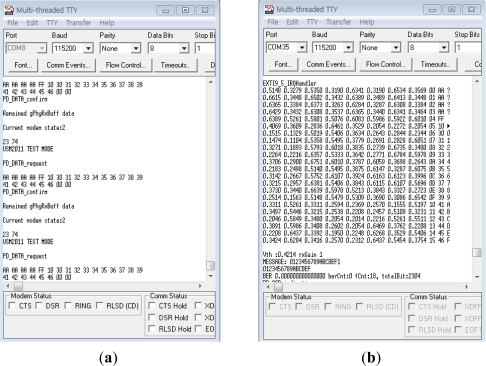
User interface for communication tests. (**a**) Transmission; (**b**) Reception.

Figure 11 shows the user interface for communication tests. The left window for transmission displays transmitted data and the right window for reception displays whether the transmitted data are received and what the received voltage level is.

**Figure 12.**
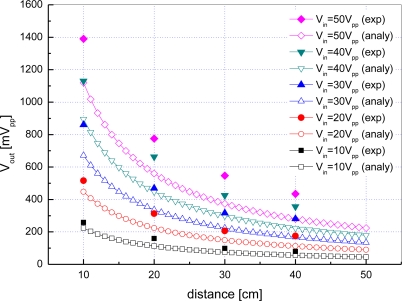
Output voltage with respect to distance and input voltage in a water tank.

Figure 12 shows a plot of the output voltage of the receiving transducer *V_out_* *versus* communication distance *d* when the input voltage of the transmitting transducer *V_in_* is set to 10 to 50 volts peak-to-peak.

Hollow symbol curves represent the analytical results obtained by Equations (1) and (6) whereas filled symbol curves stand for experimental results. As expected, according to the analytical results, *V_out_* is nearly directly proportional to *V_in_* and inversely proportional to *d*. The experimental results have the same slopes as the analytical results, but the values are a little larger than what are expected. This is because there exist strong multipath in the small-sized water tank and reflected signals combined with an original signal which make the received signal strength larger.

### 7.2. Test in a Pond

**Figure 13.**
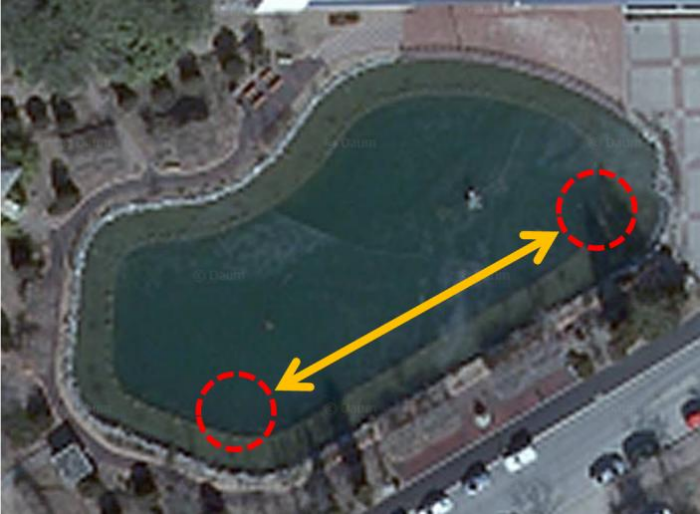
Experimental environment for the tests in a pond.

**Figure 14.**
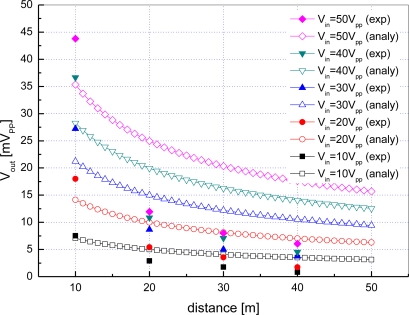
Output voltage with respect to distance and input voltage in a pond.

We have also conducted outdoor experiments in a pond shown in Figure 13, which is 60 m long, 40 m wide and 1 to 3 m deep. Transmitting and receiving transducers are submerged in 0.5 m depth from the water surface. Figure 14 shows the output voltage of the receiving transducer *V_out_* as a function of the input voltage of the transmitting transducer *V_in_* and communication distance *d*. Since the pond is very shallow when compared with its length and width, cylindrical spreading is assumed for the derivation of analytical results. According to the results, the experimental results do not exhibit a good match to the analytical ones. This is because the shape and structure of the pond is so complex that even the transmission loss model based on cylindrical spreading fails to reflect bottom topography.

For the performance evaluation of the implemented modem, we have evaluated bit error rate (BER). Fifty frames where each frame contains the physical layer payload of 255 bytes are sent over the underwater channel. That is, in total 102,000 bits are transmitted for each experiment. The distance between transmitting and receiving modems and the data rate are set to 40 m and 200 bps, respectively.

**Figure 15.**
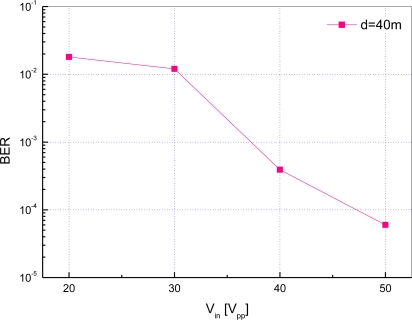
Bit error rate with respect to input voltage in a pond.

Figure 15 shows the bit error rate with respect to the input voltage *V_in_*. From the results, it is observed that the BER is improved with the increase of *V_in_* for a fixed distance. This is because *V_out_* is proportional to *V_in_*, which makes it easy to extract the original signal from noise and multipath. Meanwhile, we can achieve a BER of 3.9 × 10^−4^ at *V_in_* = 40 *V_pp_*. Since *V_out_* by experiment is equal to 4.5 *mV_pp_* at *d* = 40 *m* and *V_in_* = 40 *V_pp_* in Figure 14, we can interpret that the BER of 3.9 · 10^−4^ is achieved if the transducer at receiver side acquires *V_out_* of 4.5 *mV_pp_*.

### 7.3. Test in a River

**Figure 16.**
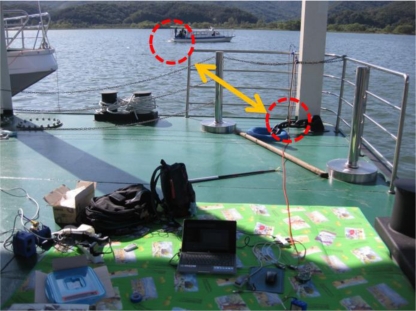
Experimental environment for the tests in a river.

Figure 16 shows a testbed for river experiments. The width of the river is 500 m and the maximum depth is about 10 m. The flow velocity in murmuring state is normally 0.1 m/s. For experiments, we put the transmitting modem at the dock and the receiving modem on the ship.

**Figure 17.**
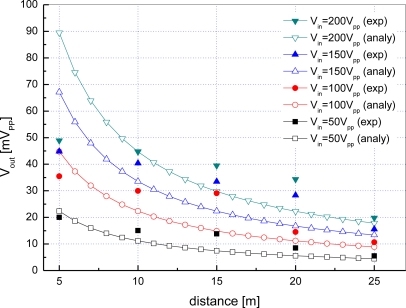
Output voltage with respect to distance and input voltage in a river.

The output voltages of the receiving transducer *V_out_* acquired by varying the distance *d* between two modems and the input voltage of the transmitting transducer *V_in_* are shown in Figure 17. With the increase of the distance, experimental results match analytical ones obtained by spherical spreading in general. But the results by experiments deviate from the expected values at some distances. This is because there are too many unexpected obstacles disturbing acoustic communication beneath the natural river surface such as topographic characteristics, waterweed and aquatic organisms. According to experiments in the river, error-free communication is possible up to 70 m at 0.2 kbps if 200 *V_pp_* is applied as the input voltage of the transmitting transducer *V_in_*.

## 8. Conclusions

In this paper, we have designed and implemented an omni-directional acoustic micro-modem based on a Cortex-M3 for use in UWSNs. The size of the developed cylindrical modem is only 70 mm in diameter and 35 mm in height. The modem is also equipped with a spherical transducer and operated by an external 14.8 volts lithium-ion battery. Physical layer functions including the issues of frame format and symbol structure were investigated and implemented fully in software and hardware. For the verification of the functionality and performance of the modem, indoor and outdoor experiments were executed in a water tank, a pond and a river. Generally, experimental results matched expected results by analysis but in some cases of river tests there were small deviations between them. According to the experiments in the river, it was possible for the modem to send data wirelessly up to 70 m with a data rate of 0.2 kbps while consuming a power of 4.5 watts. The maximum communication distance will increase if we use a higher supply voltage or a transducer having lower resonant frequency. Except for the transducer, the modem hardware is made of general purpose off-the-shelf digital and analog devices which make it possible to curtail component expenditures. Since the developed micro-modem possesses the characteristics of low-power consumption, micro size, low cost, omni-directivity and so on, it is expected that the modem can be used for various UWSN applications requiring acoustic communication.

The main contribution of this work is the development of the cylindrically shaped micro-modem guaranteeing an omni-directional beam pattern in three dimensions. From these characteristics, the modem could be utilized for underwater moving vehicles such as ROVs and AUVs, devices requiring omnidirectional property such as UWSN sink nodes, and biomimetic fish robots. As our next work, we will address the following two topics: one is to improve the performance of our modem in terms of data rate and communication distance by loading state-of-art transmission technologies such as equalization, multi-level and multi-carrier modulation and channel coding into a digital signal processor. The other is to optimize analog circuitry to enhance the hardware efficiency.

## References

[1] Crocker M. (1997). Encyclopedia of Acoustics.

[2] Kinsler L.E., Frey A.R., Coppens A.B., Sanders J.V. (1999). Fundamentals of Acoustics.

[3] Teledyne Benthos, Inc.

[4] LinkQuest, Inc.

[5] Akyildiz I.F., Pompili D., Melodia T. (2004). Challenges for efficient communication in underwater acoustic sensor networks. ACM SIGBED Rev.

[6] Akyildiz I.F., Pompili D., Melodia T. (2005). Underwater acoustic sensor networks: Research challenges. Ad Hoc Networks.

[7] Heidemann J., Ye W., Wills J., Shed A., Li Y. Research Challenges and Applications for Underwater Sensor Networking.

[8] Pandya S., Engel J., Chen J., Fan Z., Liu C. CORAL: Miniature Acoustic Communication Subsystem Architecture for Underwater Wireless Sensor Networks.

[9] Wills J., Ye W., Heidemann J. Low-Power Acoustic Modem for Dense Underwater Sensor Networks.

[10] Byeon M.-K., Kim B.-W., Jeon J.-H., Park S.-J. Design and Implementation of High-Speed Communication Modem Using Ultrasonic Sensors for Underwater Sensor Networks.

[11] Jeon J.-H., Park S.-J. Implementation of a Low-Power Acoustic Modem for Underwater Wireless Sensor Networks.

[12] Woods Hole Oceanographic Institution (WHOI).

[13] Tritech, Inc.

[14] Jeon J.-H., Won T.-H., Cho H., Park S.-J. Implementation of a Micro-Modem for Underwater Wireless Sensor Networks.

[15] Won T.-H., Cho H., Park S.-J. An Omni-Directional Underwater Acoustic Modem Based on Cortex-M3.

[16] Reson, Inc.

